# Structure Modeling of Human Tyrosyl-DNA Phosphodiesterase 1 and Screening for Its Inhibitors

**Published:** 2017

**Authors:** I.V. Gushchina, D.K. Nilov, A.L. Zakharenko, O.I. Lavrik, V.K. Švedas

**Affiliations:** Faculty of Bioengineering and Bioinformatics, Lomonosov Moscow State University, Lenin Hills 1, bldg. 73, Moscow, 119991, Russia; Belozersky Institute of Physicochemical Biology, Lomonosov Moscow State University, Lenin Hills 1 , bldg. 40, Moscow, 119991, Russia; Institute of Chemical Biology and Fundamental Medicine, Russian Academy of Sciences, Siberian Branch, Lavrentiev avenue 8, Novosibirsk, 630090, Russia; Altai State University, Lenin avenue 61, Barnaul, 656049, Russia

**Keywords:** inhibitor, docking, molecular modeling, tyrosyl-DNA phosphodiesterase 1

## Abstract

The DNA repair enzyme tyrosyl-DNA phosphodiesterase 1 (Tdp1) represents a potential molecular target for anticancer therapy. A human Tdp1 model has been constructed using the methods of quantum and molecular mechanics, taking into account the ionization states of the amino acid residues in the active site and their interactions with the substrate and competitive inhibitors. The oligonucleotide- and phosphotyrosine-binding cavities important for the inhibitor design have been identified in the enzyme’s active site. The developed molecular model allowed us to uncover new Tdp1 inhibitors whose sulfo group is capable of occupying the position of the 3’-phosphate group of the substrate and forming hydrogen bonds with Lys265, Lys495, and other amino acid residues in the phosphotyrosine binding site.

## INTRODUCTION

During DNA replication or transcription, single-strand breaks are usually introduced by topoisomerase I (Top1) in order to remove local helical tensions [1, 2]. However, various DNA damages (strand breaks, nucleobase lesions), as well as Top1 inhibition, lead to the accumulation of covalent Top1-DNA complexes with a catalytic tyrosine that is linked to the 3’-terminal phosphate [3, 4]. To maintain the native DNA structure and enable the replication process to proceed, such complexes are hydrolyzed by tyrosyl-DNA phosphodiesterase 1 (Tdp1), an important DNA repair enzyme found in humans and other eukaryotic organisms [5-8].

**Fig. 1:**
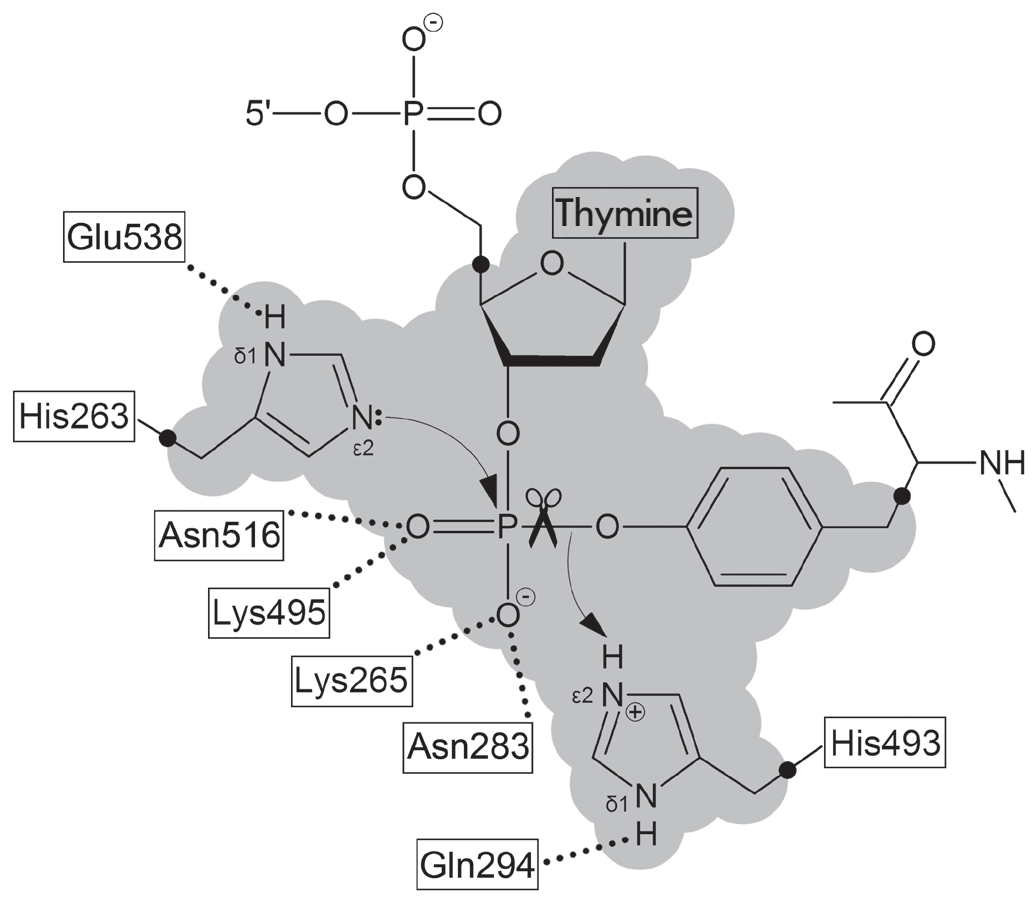
The structure of the Tdp1 active site. The Lys265, Asn283, Lys495, and Asn516 residues are involved in the binding of the substrate’s phosphate group. In the reaction mechanism, nucleophilic attack by His263 residue occurs and a proton is transferred from His493 to the leaving group. The shaded area corresponds to the QM region defined in the performed modeling of the enzyme-substrate complex.

The Tdp1 substrate is a Top1-DNA complex in which Top1 is preliminarily proteolyzed to a short peptide fragment [9]. Tdp1 exhibits broad substrate specificity, because Top1 creates nicks at various sites in the DNA backbone (although it shows preference for the thymidine 3’-phosphodiester bond) [10]. The Tdp1 active site is centrally located in a substrate-binding groove. The narrow part of the groove on one side of the active site is positively charged and involved in the binding of the DNA strand. The wider part of the groove on the other side binds a peptide fragment of the substrate. The position of the substrate’s 3’-phosphate group in the Tdp1 active center is stabilized by hydrogen bonds with the Lys265 and Lys495 residues. It is considered that carboxamide groups of Asn283 and Asn516 are also involved in the phosphate binding [11, 12]. The phosphodiester bond between the 3’-phosphate and tyrosine residue is cleaved via an SN2 mechanism, with the participation of the His263 and His493 side chains, and a transition state is formed in a trigonal bipyramidal configuration when the Nε2 atom of His263 and tyrosyl oxygen occupy apical positions at the nucleophilic attack by His263, whereas the His493 residue donates a proton to the tyrosine residue in the leaving group (*Fig. 1*) [13, 14]. The protonated state of the Nδ1 atoms of His263 and His493 is stabilized by hydrogen bonds with the Glu538 and Gln294 side chains, respectively. The deprotonation of the Nε2 atom of His263 may be forced by the close proximity of the charged amino groups of Lys265 and Lys495; and the charged state of His493, by the proximity of the Asp288 side chain.

Camptothecin and its derivatives (irinotecan, topotecan) cause the formation of irreversible covalent Top1-DNA complexes and are, therefore, used to inflict DNA damage on cancer cells [3]. The suppression of the elimination of such complexes by Tdp1 inhibitors is a promising way with which to enhance the antitumor effect of camptothecins, which is confirmed by the fact that *TDP1*-deficient cells are sensitive to chemotherapy [15-17]. While there are several compounds known to suppress enzyme activity, drug development based on Tdp1 inhibitors remains far from a preclinical or clinical stage. For instance, the vanadate ion VO43-, forming a coordinate bond with His263 and resembling the transition state of the reaction, was used to study the catalytic mechanism and to obtain crystal Tdp1 complexes with various oligonucleotides and peptide fragments [10, 13]. Tdp1 inhibitors were detected by *in vitro *screening of low-molecular-weight compounds, including steroid derivatives [18], indenoisoquinolines [19, 20], phosphotyrosine mimetics [21], thioxothiazolidinones [22], benzopentathiepines [23], and diazaadamantanes [24]. The above-mentioned compounds presumably compete for the substrate binding site, though the structures of the enzyme-inhibitor complexes are unknown, and the specific interactions between these molecules and active site residues are still to be uncovered. A molecular docking investigation of the interactions between several inhibitors and Tdp1 led to contradictory results which poorly correlated with experimental data on the inhibitory effect of the compounds [25, 26]. This suggests that protein models built on the basis of crystal structures need to be elaborated and optimized. In some studies, the reaction mechanism and molecular environment were not taken into account when estimating the ionization states of the histidine [22] and lysine [18, 27] side chains in the active site: that questions the reliability of the modeling. Obviously, a high-quality model of human Tdp1 which takes into account the structural features of the active site is needed to simulate the binding of potential inhibitors. The goal of the present study was to build a molecular model of Tdp1 using hybrid methods of quantum and molecular mechanics, as well as to verify its validity for virtual screening for competitive inhibitors.

## EXPERIMENTAL SECTION

**Protein structure modeling**

The molecular model of human Tdp1 was built on the basis of the 1nop crystal structure (chains A, C, D) [14]. The coordinates of the missing loops in the protein structure were predicted with the Swiss-PDBViewer 4.1 program (which implements structure superimposition) [28] and ModLoop web server (predicts the position of the missing heavy atoms) [29]. The coordinates of the loop 425-434 were transferred from the 1qzq structure following its superimposition onto 1nop, and the coordinates of the loop 560-567 missing in all Tdp1 crystal structures were predicted from the amino acid sequence.

Next, the enzyme-substrate complex of Tdp1 was modeled using the AmberTools 1.2 (http://ambermd.org) and Amber 12 [30, 31] packages installed on the MSU supercomputer [32]. The substrate molecule was constructed based on a structural analogue from 1nop (covalent complex vanadate-oligonucleotide-peptide), by replacing the vanadium atom with phosphorus. Parameters from the AMBER parameter database [33] were used to provide a molecular mechanical description for the phosphotyrosine moiety of the substrate molecule. The remaining portion of the substrate and the protein were described by the *ff99SB *force field [34]. Hydrogen atoms were added to the structure of the enzyme-substrate complex, and, then, it was placed in a water box (TIP3P solvent model, minimum distance of 12 Å between the protein and the box’s edge). Chlorine ions were added into the box to neutralize the positive net charge caused by the ionogenic groups of the protein and the substrate. The energy minimization of the obtained system was performed in two stages. At the first stage (2,500 steps of the steepest descent algorithm followed by 2,500 conjugate gradient steps), the protein and substrate coordinates were kept fixed by positional constraints of 2 kcal/(mol∙Å2) on heavy atoms. At the second stage (5,000 steepest descent steps followed by 5,000 conjugate gradient steps), the system was partitioned into quantum mechanics (QM) and molecular mechanics (MM) regions. The QM region consisting of a fragment of the substrate and the side chains of His263 and His493 (see *Fig. 1*) was described using the semi-empirical Hamiltonian RM1 [35, 36] and linker atoms at the region boundaries. A PME (Particle Mesh Ewald) approach and periodic boundary conditions were chosen in computing long-range electrostatic interactions.

A search for binding pockets in the obtained Tdp1 structure was performed using the fpocket 2.0 and pocketZebra software [37, 38], with cavities identified as clusters of alpha spheres (spheres that are in contact with four atoms and do not contain internal atoms). To identify small cavities, the minimum number of alpha spheres in a cavity was reduced from 35 to 30, and the maximum distance between alpha spheres at a clustering step was also reduced from 2.5 to 2.4 Å. Hydrogen atoms were not taken into account during the search for cavities.

**Virtual screening**

Virtual screening for Tdp1 inhibitors was carried out among low-molecular-weight compounds from the Vitas-M commercial library (http://www.vitasmlab. com). The protonation and structure optimization of compounds was performed as described previously [39]. Compounds containing a sulfo group and in conformity with the rule of three (molecular weight < 300, log *P *≤ 3, hydrogen bond donors ≤ 3, hydrogen bond acceptors ≤ 3, rotatable bonds ≤ 3) [40, 41] were selected from the library using the ACD/SpectrusDB 14.0 program (http://www.acdlabs.com). The substrate and water molecules were removed from the obtained model of Tdp1 enzyme-substrate complex, and an energy grid box (map of interaction potential) overlapping the active site was generated through the Lead Finder 1.1.15 program [42, 43]. Next, the molecular docking of the compounds into the Tdp1 active site was performed using a genetic algorithm in “extra precision” mode. The resulting structures of the complexes with inhibitors were optimized according to the procedure applied to the Tdp1 enzyme-substrate complex. The QM-region included an inhibitor molecule and the side chains of His263 and His493, and the molecular-mechanical parameters of the inhibitors were taken from the *GAFF *force field [44]. The visualization of predicted poses was performed using the VMD 1.9.2 software [45].

**Enzyme activity assay**

The recombinant human Tdp1 protein was expressed in *Escherichia coli *and extracted according to the earlier described procedure [46]. The plasmid pET 16B Tdp1 was kindly provided by Dr. K.W. Caldecott (University of Sussex, United Kingdom). The enzyme was purified by chromatography with nickel sorbent NTA-Ni^2+^-Sepharose CL-6B, and, then, the final purification was done with phosphocellulose P-11. A previously constructed biosensor 5’-(5,6 FAM-aac gtc agg gtc ttc c-BHQ1)-3’, where FAM is a fluorophore and BHQ1 is a fluorescence quencher, was used for measurements of enzyme activity [23, 47]. The Tdp1 activity was monitored by detecting the release of 3’-terminal substituent BHQ1 under the following conditions: 50 mM Tris-HCl, pH 8.0, 50 mM NaCl, 7 mM β-mercaptoethanol, 50 nM biosensor, 1.3 nM Tdp1, 26°C. The reaction rate at different concentrations of compounds STK370528 (Sigma-Aldrich) and STK376552 (Vitas- M Laboratory, Ltd) was measured using a POLARstar OPTIMA fluorimeter (BMG LABTECH, Germany). The measurements were conducted in two independent experiments. The IC_50_ values (the inhibitor concentration required to reduce the enzyme activity by 50% [48]) were determined using the MARS Data Analysis 2.0 program (BMG LABTECH).

## RESULTS AND DISCUSSION

**Protein model**

To construct a molecular model of human Tdp1 that could be used to screen for its competitive inhibitors, it was necessary to select an appropriate crystal structure of the enzyme, take into account the ionization of catalytically important amino acid residues, and reproduce the conformations of these residues that allow for an optimal interaction with the substrate. The Protein Data Bank contains structures of the Tdp1 apo form (PDB ID 1jy1, 1qzq), as well as complexes with various transition state analogues (1mu7, 1mu9, 1nop, 1rff, 1rfi, 1rg1, 1rg2, 1rh0, 1rgt, 1rgu). A complex with the closest substrate analogue – 1nop – in which vanadate is covalently bound to the catalytic His263 residue was selected as the initial structure for modeling. Through the replacement of the vanadium atom with phosphorus, the starting substrate structure was obtained: the oligonucleotide 5’-GTT-3’ linked to the peptide KLNYL via a tyrosine side chain.

**Table 1:** Interactions of the 3’-terminal phosphate group of the substrate with the active site residues in the starting and optimized models of human Tdp1.

Interaction	Distance (Å)	
	Starting model	Optimized model
PO_4_^-^:P ∙∙∙ His263:NE2	2.0	2.7
PO_4_^-^:O_bridging_ ∙∙∙ His493:NE2	2.6	2.6
PO_4_^-^:O ∙∙∙ Lys265:NZ	2.8	2.7
PO_4_^-^:O ∙∙∙ Lys495:NZ	2.8	2.7
PO_4_^-^:O ∙∙∙ Asn283:ND2	3.0	2.8
PO_4_^-^:O ∙∙∙ Asn516:ND2	3.2	3.0

An important modeling step was the reconstruction of the missing loops 425-434 and 560-567, as a protein structure without chain breaks was required for further optimization. Hydrogen atoms were added to the Tdp1 structure with reconstructed loops; a hydrogen atom was attached to the Nδ1 atom of the His263 side chain, and the His493, Lys265, and Lys495 side chains were taken to be charged. The optimization of the coordinates of the substrate and those of the added hydrogen atoms was done in two stages. At the first stage, a molecular-mechanical minimization was performed to remove the largest strains in the system. At the second stage, the semi-empirical Hamiltonian RM1, whose efficiency was demonstrated in simulations of biomolecules [49, 50], was used to describe the interactions between the substrate and the catalytic residues His263 and His493 more precisely. The most important interatomic distances in the active site of the starting and optimized Tdp1 models are listed in *Table 1*. The initial position of phosphate atoms in the starting model corresponds to the coordinates of vanadate in a complex with the enzyme, resembling the transition state. Through the structure optimization, the phosphate adopts a tetrahedral configuration and the distance between phosphorus and His263 increases from 2.0 to 2.7 Å, which corresponds to their disposition in the ground state of the active site. Hydrogen bonding of the phosphate group with other residues does not undergo significant changes. This demonstrates that in both the ground state and transition state the Asn283 and Asn516 side chains participate in the substrate binding and, together with the charged amino groups of Lys265 and Lys495, form a hydrogen-bonding network with the 3’-phosphate group. A Tdp1 model for the docking of small molecules was obtained by removing the substrate from the optimized structure, where the orientations of the active site residues could provide multiple interactions with competitive inhibitors.

**Fig. 2:**
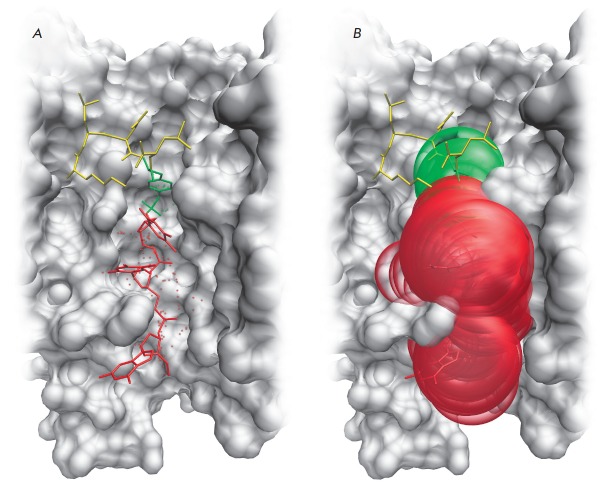
The substrate-binding groove in the human Tdp1 model. (*A*) The interaction of the substrate molecule with the oligonucleotide and phosphotyrosine binding sites. The oligonucleotide is shown in red, phosphotyrosine is shown in green, and the rest of the peptide is shown in yellow. Cavities are labeled with points corresponding to the centers of alpha spheres. (*B*) The localization of alpha spheres in the oligonucleotide and phosphotyrosine binding sites.

An analysis of the substrate-binding groove surface in the Tdp1 model allowed us to identify binding sites for potential inhibitors. There are two distinct binding cavities; one for the phosphotyrosine and a second for the oligonucleotide, with the Asn516 and His263 side chains located at the boundary between them (*Fig. 2*). The oligonucleotide-binding cavity is a large region which has a total surface area of 666 Å2. Among the amino acid residues positioned in this region are the residue pairs Ser400-Ser518 and Ser403-Ala520 involved in the binding of the second and third phosphate groups from the 3’-terminus. The phosphotyrosine-binding cavity is substantially smaller (206 Å2), but all the key active site residues participate in its formation: His263, His493, Lys265, Lys495, Asn283, Asn516, as well as the Tyr204, Pro461 and Trp590 residues involved in hydrophobic contacts.

**Fig. 3:**
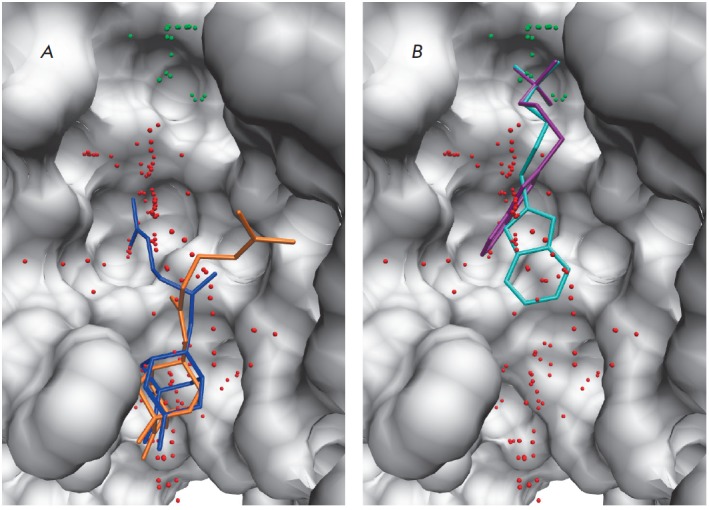
Interactions of inhibitors with the substrate-binding groove in the human Tdp1 model. (*A*) The binding of diazaadamantane derivatives. (*B*) The binding of the sulfo-substituted derivatives STK370528 and STK376552. The oligonucleotide and phosphotyrosine cavities are labeled with red and green alpha spheres, respectively.

Most of the known Tdp1 inhibitors are deprived of negatively charged moieties. Therefore, it is quite possible that the phosphotyrosine cavity, adapted to accommodate the 3’-terminal phosphate, does not participate in the binding of these compounds. This assumption is confirmed by a modeling of inhibitor binding using molecular docking. So, diazaadamantane derivatives, whose inhibitory properties were recently reported [24], are localized in the oligonucleotide region of the active site upon simulation of their binding using the Tdp1 model. The tricyclic moiety of these inhibitors occupies the site of the third ribose residue from the 3’-terminus, while an extended hydrophobic substituent is oriented towards the phosphotyrosine binding site, but does not interact with it (*Fig. 3A*).

**Inhibitor screening**

The presence of a cluster of the conserved Lys265, Lys495, Asn283, and Asn516 residues in the phosphotyrosine binding site makes possible an effective electrostatic interaction between the enzyme and substrate and may be an important structural factor in the binding of competitive inhibitors containing an appropriate charged group. A sulfo group, SO_3_^-^ , might serve as a functional group of that type, being a structural analogue of phosphate. To verify this assumption, sulfonic acids and their salts (71 compounds) were retrieved from a library of low-molecular-weight compounds conforming to the rule of three that specifies the ranges of physicochemical parameters of the molecular fragments (small molecules used in primary screening and subsequent structure optimization). The compounds were docked into the Tdp1 model active site and examined for their ability to form hydrogen bonds with Lys265, Lys495, Asn283, Asn516, as well as other interactions with the DNA and peptide binding sites.

**Table 2:** Compounds selected by virtual screening as human Tdp1 inhibitors.

	Chemical structure	Δ^Gcalc^(kcal/mol)	ΔG^recalc^(kcal/mol)	IC_50_ (μM)
STK370528	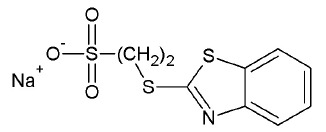	-7.5	-8.7	83±24
STK376552	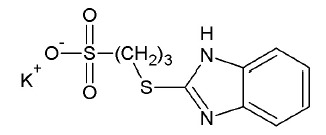	-8.4	-8.0	686±14

As a result of the screening, we selected the most promising inhibitors, STK370528 and STK376552, in which the sulfo group was attached to a heterocyclic moiety via a thioether linker (*Table 2*, (*Fig. 3B*). The conformations of amino acid residues that interact with STK370528 and STK376552 in the obtained enzymeinhibitor complexes were subsequently optimized using the RM1 Hamiltonian. Re-docking into the refined protein models revealed that STK370528 was a more effective inhibitor and had higher binding energy Δ*G*recalc (see data in *Table 2*).

**Fig. 4:**
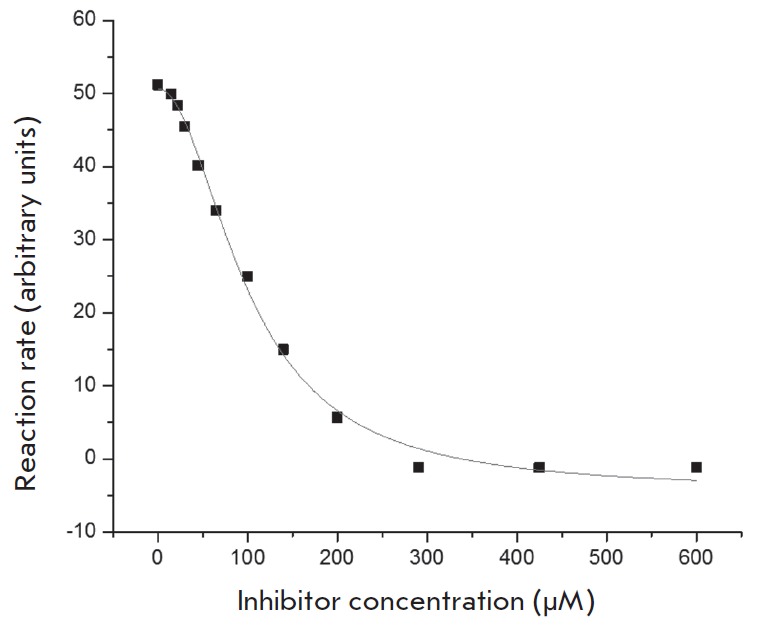
The dependence of the Tdp1-catalyzed reaction rate on the concentration of the inhibitor STK370528.

For the experimental testing of the inhibitory properties of the compounds against the recombinant form of human Tdp1, we applied a biosensor (an oligonucleotide containing a fluorophore at the 5’-end and a fluorescence quencher at the 3’-end) that enables measurement of enzyme activity in real time. The method is based on the ability of Tdp1 to remove various large adducts from the 3’-end of DNA [17], including the fluorescence quencher BHQ1 (Black Hole Quencher 1) [51]. Upon BHQ1 removal by the enzyme, the intensity of the 5’-terminal fluorophore emission depends on the amount of cleaved substrate. *Figure 4*shows a typical plot of the reaction rate as a function of the STK370528 concentration. The IC_50_ values were 83 μM for STK370528 and 686 μM for STK376552. Thus, the experimental study confirmed the conclusions of molecular modeling and showed that the selected compounds were Tdp1 inhibitors that suppress enzyme activity in the micromolar concentration range.

**Fig. 5:**
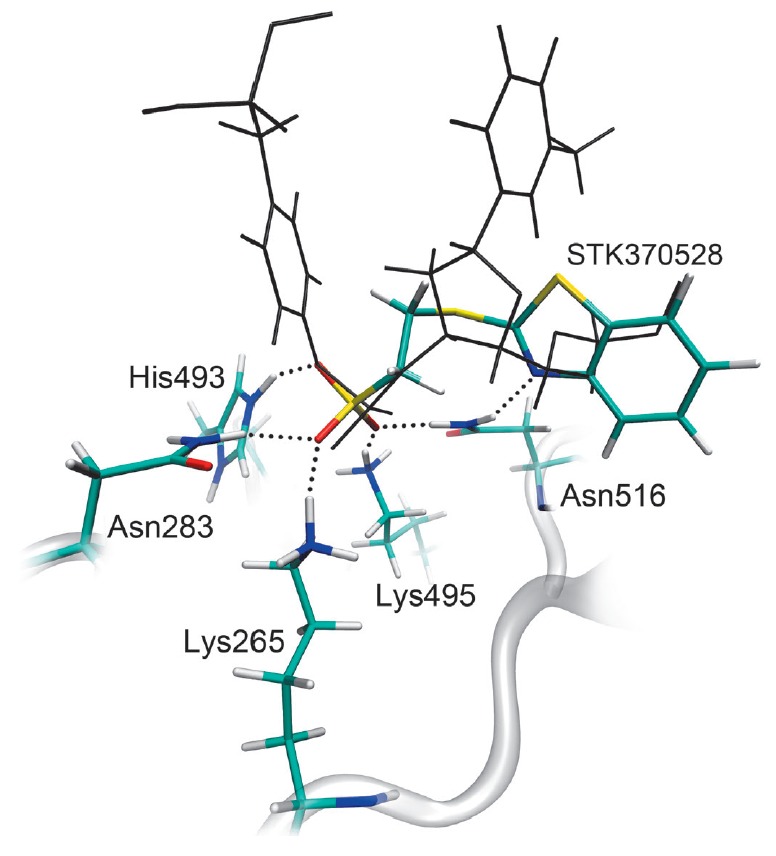
The position of the inhibitor, STK370528, in the active site of the molecular model of human Tdp1. Dotted lines indicate hydrogen bonds important for the stabilization of the sulfo group position. The gray color denotes the substrate coordinates in the model of the enzyme-substrate complex.

The sulfo group of the inhibitors is capable of occupying the position of the 3’-phosphate group of the substrate and can form hydrogen bonds with the amino acid residues Lys265, Lys495, Asn283, Asn516, and His493, which constitute the phosphotyrosine binding site (*Fig. 5*). The location of the heterocyclic moiety in the oligonucleotide binding site leads to additional interactions. In the case of STK370528, a benzothiazole group forms a hydrogen bond with Asn516 and hydrophobic contacts with the Ala520 and Ala521 side chains. A flexible linker in the inhibitor structure provides a connection between groups located in different regions of the Tdp1 active site. The linker in STK376552 is elongated by one methylene unit, which disrupts interactions with Ala521 and Asn516 and decreases the inhibitory activity of this compound compared to STK370528.

Electrostatic interactions with the charged residues Lys265, Lys495, and His493 play an important role in the binding and orientation of inhibitors in the Tdp1 active site. In the case of uncharged sulfonates (phenyl and methyl esters of STK370528), the efficiency of interaction with the active site residues is reduced as confirmed by a number of different inhibitors’ orientations upon simulation of their binding in the enzyme active site. Modeling of the binding of indenoizoquinoline sulfonates which had been previously considered to be potential inhibitors but exhibited no activity against Tdp1 [25], has also shown that an esterified sulfo group cannot mediate interactions with the phosphotyrosine binding site.

## CONCLUSIONS

The present study shows that the constructed molecular model of the DNA repair enzyme Tdp1, taking into account the structural features of the active site, adequately describes the binding of small molecules and makes possible a selection of substrate-competitive inhibitors through virtual screening. Based on a detailed analysis of intermolecular interactions, we selected from the computer library of potential inhibitors the sulfonates STK370528 and STK376552, which are capable of suppressing enzyme activity in the micromolar concentration range. The structural organization and localization of the oligonucleotide- and phosphotyrosine-binding sites in the substrate-binding groove were shown to be important factors to be considered when developing new Tdp1 inhibitors.

## Abbreviations

Tdp1: tyrosyl-DNA phosphodiesterase 1
Top1: topoisomerase I
QM: quantum mechanics
MM: molecular mechanics
